# **NeOProM: Ne**onatal **O**xygenation **Pro**spective **M**eta-analysis Collaboration study protocol

**DOI:** 10.1186/1471-2431-11-6

**Published:** 2011-01-17

**Authors:** Lisa M Askie, Peter Brocklehurst, Brian A Darlow, Neil Finer, Barbara Schmidt, William Tarnow-Mordi

**Affiliations:** 1 NHMRC Clinical Trials Centre, University of Sydney, (Parramatta Road), Camperdown, (2050), Australia; 2 National Perinatal Epidemiology Unit (NPEU), University of Oxford, (Old Road Campus), Oxford, (OX3 7LF), UK; 3 Christchurch School of Medicine, University of Otago, (Riccarton Avenue), Christchurch, (8140), New Zealand; 4 Division of Neonatology, University of California San Diego (UCSD) Medical Center, (West Arbor Drive), San Diego, (92103), USA; 5 Children's Hospital of Philadelphia, University of Pennsylvania, (Spruce Street), Philadelphia, (19104), USA; 6 Neonatal Trials Group, McMaster University, (Concession Street), Hamilton, (L8V 1C3), Canada; 7 Westmead Hospital, University of Sydney, (Cnr Hawkesbury and Darcy Roads), Westmead, (2145), Australia; 8 Children's Hospital at Westmead, University of Sydney, (Cnr Hawkesbury Road and Hainsworth Street), Westmead, (2145), Australia

## Abstract

**Background:**

The appropriate level of oxygenation for extremely preterm neonates (<28 weeks' gestation) to maximise the greatest chance of survival, without incurring significant morbidity, remains unknown. Infants exposed to lower levels of oxygen (targeting oxygen saturations of <90%) in the first weeks of life are at increased risk of death, cerebral palsy, patent ductus arteriosus, pulmonary vascular resistance and apnoea, whilst those maintained in higher levels of oxygen (targeting oxygen saturations of >90%) have been reported to have greater rates of morbidity including retinopathy of prematurity and chronic lung disease. In order to answer this clinical dilemma reliably, large scale trial evidence is needed.

**Methods/Design:**

To detect a small but important 4% increase in death or severe disability in survivors, over 5000 neonates would need to be recruited. As extreme prematurity affects 1% of births, such a project undertaken by one trial group would be prohibitively lengthy and expensive. Hence, the Neonatal Oxygenation Prospective Meta-analysis (NeOProM) Collaboration has been formed. A prospective meta-analysis (PMA) is one where studies are identified, evaluated, and determined to be eligible before the results of any included studies are known or published, thereby avoiding some of the potential biases inherent in standard, retrospective meta-analyses. This methodology provides the same strengths as a single large-scale multicentre randomised study whilst allowing greater pragmatic flexibility. The NeOProM Collaboration protocol (NCT01124331) has been agreed prior to the results of individual trials being available. This includes pre-specifying the hypotheses, inclusion criteria and outcome measures to be used. Each trial will first publish their respective results as they become available and the combined meta-analytic results, using individual patient data, will be published when all trials are complete. The primary outcome to be assessed is a composite outcome of death or major disability at 18 months - 2 years corrected age. Secondary outcomes include several measures of neonatal morbidity. The size of the combined dataset will allow the effect of the interventions to be explored more reliably with respect to pre-specified patient- and intervention-level characteristics.

**Discussion:**

Results should be available by 2014.

## Background

Extreme prematurity of less than 28 weeks' gestation affects approximately 1% of births [1]. Although 80% of these infants are discharged home alive [2], they often sustain severe morbidity [3], including chronic lung disease, poor growth, respiratory illness, hospital re-admissions, visual deficits, cerebral palsy, sensori-neural disability and cognitive, educational and behavioural impairment [4]. Recent studies have highlighted specific health issues former very preterm infants may face in later life, including an increased risk of cardiovascular disease and hypertension, impaired glucose tolerance, impaired renal function and abnormal respiratory function [5-9]. Very preterm infants account for a high proportion of the costs and disability from neonatal intensive care [10]. Reducing these morbidities would enhance quality of life for these infants and benefit their families and communities [11].

Oxygen is the most common therapy used in the care of very preterm infants. It has been associated with significant improvements in neonatal survival and disability [12]. However, preterm infants are highly sensitive to the harmful biochemical and physiological effects of supplemental oxygen. Toxic oxygen radicals are increased in hyperoxaemia [13] and in re-oxygenation after hypoxaemia. Preterm infants are vulnerable to oxidative stress because they lack antioxidant protection [13] from plasma radical scavengers such as beta- carotene, antioxidant enzymes, such as glutathione peroxidase, and their red cells lack superoxide dismutase.

Hyperoxaemia can constrict or obliterate vessels in an immature eye and brain, causing ischaemic injury [13]. Of survivors born at less than 28 weeks' gestation, 49% have retinopathy of prematurity (ROP), 12.4% have severe (Grade III or IV) ROP, 86% of these have surgery [2], but about 10% of those treated become blind. New recommendations have resulted in more infants with severe ROP having laser surgery [14].

High inspired oxygen contributes to bronchopulmonary dysplasia [15,16]. Oxidative damage to premyelinating oligodendrocytes in cerebral white matter is proposed as a mechanism of periventricular leukomalacia [17] which has been correlated with cerebral palsy. In preterm infants, oxygen reduces cerebral blood flow velocity independently of the effects of hypocapnia or hypotension [18].

Hyperoxaemia in the first eight days has been associated with twice the odds of cerebral palsy at 2 years [19]. In this study, the adjusted odds of cerebral palsy increased eightfold for infants with the highest versus the lowest quintile of exposure to hyperoxaemia, indicating a dose-response effect. Importantly, hyperoxaemia was defined as arterial oxygen above 60 mm Hg, in contrast with the long accepted upper limit of 80 mm Hg [20,21].

Less exposure to oxygen is a simple strategy that could reduce oxidative stress and tissue injury and prevent morbidity in very preterm infants. In healthy preterm infants breathing air, arterial oxygen saturation (SpO_2_) is 85-98%. However, for infants requiring supplemental oxygen, the optimum range of arterial oxygen to minimise organ damage, without causing hypoxic injury, remains unknown.

### Summary of existing evidence

The first case of ROP was reported in 1942. By 1954, ROP had blinded about 10,000 infants [22,23]. In 1954-56, three randomised trials, enrolling 341 infants, proved that breathing unrestricted concentrations of inspired oxygen was a major cause of ROP [24]. As arterial oxygen levels were not able to be measured, the concentration of inspired oxygen could not be targeted to meet each baby's needs. Following these findings, premature infants were restricted to breathing less than 40% inspired oxygen in order to prevent ROP. In the next 20 years over 150,000 premature babies died of hypoxic respiratory failure [12,25-27]. It is estimated that for every infant whose sight was saved, 16 died [12,22,24] and many others developed spastic diplegia [26].

The Supplemental Therapeutic Oxygen for Prethreshold Retinopathy of Prematurity (STOP ROP) trial [28] used pulse oximetry to target lower (89-94%) or higher (96-99%) SpO_2 _in 649 preterm infants with pre-threshold ROP. The higher range caused more adverse respiratory events, including pneumonia, chronic lung disease requiring oxygen and diuretic therapy. There was no statistically significant difference in the rate of progression to threshold ROP. In the Benefits of Oxygen Saturation Targeting (BOOST) trial [29], 358 infants born at less than 30 weeks' gestation were randomly assigned, from 3 weeks or more after birth until they breathed air, to target a SpO_2 _range of either 91-94% or 95-98%. This trial found no evidence that higher SpO_2 _targeting improved growth or development, but it did increase days of oxygen therapy and use of health care resources. Masked, adjusted oximeters were used in this trial so that some were adjusted to display masked values 2% lower than actual SpO_2_, and others displayed masked values 2% higher. Staff were unaware of actual SpO_2 _and targeted a masked range of 93-96%. The authors concluded that further large randomised trials were needed to determine how targeting different SpO_2 _levels from the day of birth affects ROP, chronic lung disease, growth, disability and mortality [22,29].

An early cohort study, reported in 1977, was unable to establish a relationship between arterial oxygen tension (PaO_2_) and retinopathy [30]. A PaO_2 _range of 50-80 mm Hg became widely accepted as an appropriate level to target [20,21,31], but was based on professional consensus rather than evidence. A later study confirmed that ROP occurred more often with longer periods of time with a PaO_2 _above 80 mm Hg [32], but did not determine if another limit was safer. Oximeters measuring functional SpO_2 _display values about 1.5% higher than those measuring fractional SpO_2 _[33]. Normal fetal oxygen saturation is 70-80% [34]. In transposing oxygen tensions of 50-80 mm Hg into equivalent arterial oxygen saturation, most clinicians have targeted functional SpO_2 _90-95% (the mid range of what is considered physiological) with a minimum of 85% [35].

In a more recent cohort study, Tin et al [34] showed that lower SpO_2 _correlated with improved short term respiratory and growth outcomes in infants born at less than 28 weeks' gestation. Babies in the neonatal intensive care units (NICU) targeting SpO_2 _70-90% had ROP surgery less often than those in the NICUs targeting SpO_2 _88-98% (6.2% *v *27.2%, 80% relative risk reduction (RRR), p < 0.01). Survivors were ventilated less often (13.9 *v *31.4 days), fewer needed oxygen at 36 weeks' postmenstrual age (18% *v *46%, 61% RRR), and fewer were below the 3rd centile for weight at discharge (17% *v *45%, 62% RRR) (all p < 0.01) while survival (52% *v *53%) and cerebral palsy (15% *v *17%) at one year were similar [34].

Anderson et al [35] reported less Grade III/IV ROP (2.4% *v *5.5%, p < 0.001) and less ROP surgery (1.3% *v *3.3%, 61% RRR, p < 0.037) in NICUs with functional SpO_2 _upper limits of ≤92% vs >92%. Sun and colleagues [36] studied 1544 infants weighing < 1000 g in NICUs with upper SpO_2 _limits of ≤95% *v *>95%. NICUs with ≤95% upper limits had less Grade III ROP (10% *v *29%), surgery (4% *v *12%, 67% RRR), chronic lung disease (27% *v *53%, 49% RRR) (all p < 0.001) and similar mortality (17% *v *24%). Chow et al [37] found that a functional SpO_2 _of 83-90% was associated with less Grade III/IV ROP than an SpO_2 _of 90-98% in historical controls. This study found that from 1998 to 2001, severe ROP fell from 12.5% to 2.5% (80% RRR, p = 0.01) and ROP surgery declined from 7.5% (6/80) to zero (0/188) (100% RRR, p = 0.0006). These cohort studies thus suggest that lower SpO_2 _may reduce ROP surgery by 61-100%; chronic lung disease by 49-61%; and poor growth by 62%. Effects on mortality and long term sensori-neural outcome remain unknown and could be beneficial or harmful.

There are two opposing concerns. Less inspired oxygen (targeting SpO_2 _< 90%) may increase patent ductus arteriosus, pulmonary vascular resistance and apnoea, and impair survival and neuro-development [38-40]. More inspired oxygen (targeting SpO_2 _> 90%) may increase severe ROP and chronic lung disease [16,34-37]. After recent studies [34-37], more NICUs are adopting a lower SpO_2 _policy. This trend may increase before the risks and benefits are determined. The disastrous mistakes of the 1950s [16,18,23,25,27] show how rapidly opinions can shift, destroying the chance of obtaining reliable evidence.

Worldwide demands to resolve the dilemma are intensifying. In 2003, an eminent international group of over 30 trialists, bio-statisticians, neonatologists, ophthalmologists and developmental paediatricians was convened to conduct the NeOProM (Neonatal Oxygenation Prospective Meta-analysis) Collaboration. In December 2003, the NeOProM project was outlined in a commentary in *Pediatrics *[41].

There are five trials which are currently in progress to assess this question (see Table 1). Each of these trials will recruit between 300 and 1300 babies. However, none individually will be able to exclude the possibility that the expected valuable short term benefits associated with giving babies less oxygen are not associated with a small but significant 4% increase in death or serious neurosensory disability in survivors, from an average baseline of 42%. Reliably excluding a small, but clinically important, difference in death or severe disability of 4% requires over 5000 infants, which no single trial is able to recruit in a timely fashion. For example the Australian BOOST II trial which plans to recruit approximately 1200 infants will be able to exclude a difference of 8% (from 37% to 45% or from 37% to 29%) in the major composite outcome of death or severe disability in survivors.

**Table 1:** Eligible trials collaborating in the NeOProM initiative at December 2009

Trial acronym	Country	Planned n	Recruitment start date	Recruitment finish date	Follow-up data finalised	Planned date of publication
SUPPORT	USA	1310	April 2005	April 2009	April 2011	May 2010 (short term outcomes) Dec 2011 (longer term follow-up)
BOOST II	Australia	1200	Mar 2006	Dec 2010	Dec 2012	May 2013
BOOST-NZ	NZ	320	Sep 2006	Dec 2009	Dec 2011	Dec 2012
COT	Canada	1200	Jan 2007	Jul 2010	Dec 2011	Jun 2012
BOOST II-UK	UK	1200	Sep 2007	Feb 2011	Feb 2013	Sep 2013

For this reason, the Principal Investigators of the participating trials have pledged their support for a prospective meta-analysis (PMA) of individual patient data (IPD) from each of these studies. These five trials are sufficiently similar in terms of the population enrolled, interventions tested and outcomes measured to allow the collection and combination of IPD from each trial into a large, core, common dataset. Combining the data from several trials of similar design using PMA methodology differs from a standard meta-analysis of trial results in several important ways.

A prospective meta-analysis (PMA) is a meta-analysis where studies (usually randomised controlled trials) are identified, evaluated, and determined to be eligible before the results of included studies are known or published. This methodology can help avoid some of the potential biases inherent in standard, retrospective meta-analyses. These can include publication bias, selection bias of subjects and trials; and bias due to *post hoc *selection of study questions, eligibility criteria, outcome definitions or subgroups [42,43].

The key features of PMA are to prospectively define and clearly specify the objectives, research question(s), specific aims, hypotheses, subject eligibility criteria, subgroups, predictors, outcomes (primary and secondary) and the analysis plans of eligible studies in advance of knowing or publishing individual trial results [42]. PMA provides more reliable estimates of treatment effects through prospectively planned combined analysis of large-scale randomised controlled trials. In addition to having greater power to detect meaningful modest differences in less frequent, clinically important outcomes, PMA provides adequate power to evaluate events in important subgroups underrepresented in smaller trials. Thus, PMA provides the same strengths as a single large-scale multicentre randomised study.

Another advantage is that PMA provides greater, pragmatic flexibility in achieving the objectives of a single mega-trial. Through prospectively planned combined analysis of large, randomised trials, PMA accommodates funding agency variations, reduces costs to an individual funding agency for a mega-trial, whilst providing the same strengths and benefits of a single large randomised study. In this regard, PMA sets an important precedent for future large neonatal trials. PMAs encourage uniformity in common protocol items amongst trials, including data collection and outcome definitions, whilst permitting flexibility in pre-specified protocol details and funding regulations. To protect the integrity of each individual trial, the main PMA results are published only after the principal results of each individual trial have been published. This methodology also has the flexibility to allow questions to be added after the PMA protocol has been developed provided the additional studies or questions are chosen in a manner masked to the results of included trials [42].

By establishing collaboration between trialists of the eligible studies, it is possible to collect individual patient data (IPD) and incorporate it into the meta-analysis. Using data collected from each individual within a trial, rather than relying on aggregate data from each trial, can improve the power and scope of the meta-analysis. In particular, a meta-analysis using IPD can enable more flexible and detailed subgroup analyses [44,45].

This will be the first-ever neonatal prospective meta-analysis. However, the methodology has been used extensively in other areas of health care, particularly in cardiovascular disease [43] and cancer [46]. Large PMAs such as the Blood Pressure Lowering Treatment Trialists Collaboration recently published [47] demonstrate how this methodology can be used very effectively to assess treatment effects in specific subgroups.

The **Ne**onatal **O**xygenation **Pro**spective **M**eta-analysis (NeOProM) Collaboration will be coordinated in Sydney, Australia. The Principal Investigators of each of the trials involved in NeOProM will be members of the Collaboration's Management Committee. Thus, this is an opportunity to adapt the methodologies of prospective meta-analysis and individual patient data meta-analysis, already well-established in other health care fields, for use in answering important neonatal questions.

### Objectives

The primary question to be addressed by this study is: does targeting a lower oxygen saturation range in extremely preterm infants from birth or soon after, increase or decrease the composite outcome of death or major disability in survivors by 4% or more?

### Hypotheses

Compared with a functional oxygen saturation level (SpO_2_) of 91-95%, targeting SpO_2 _85-89% within 24 hours of birth is associated with <4% absolute risk difference from 42% [4,10] to 46% or from 42% to 38% (10% relative risk increase or reduction (RRR)) in mortality and major disability by 2 years corrected age (defined as gestational age plus chronological age).

### Sample size

A total sample size of 5230 (including infants from the SUPPORT, BOOST II Australia, BOOST-NZ, BOOST II UK, COT trials) (see Table 1 for trial details) would have a 80% power to detect a 4% difference in the primary outcome: death or major disability. The precision of the combined sample size will ensure that a 4% increase in death or major disability could be detected (for example from 42% to 46%), with 95% confidence that the true result was an increase in this outcome from 42% to between 43.7% and 48.7% (RR1.10, 95% CI 1.04-1.16).

## Methods/Design

The Principal Investigators of all eligible trials were approached and have agreed to participate in the NeOProM Collaboration and provide the relevant individual patient data upon completion of their respective trials. A common data collection form, coding sheet and detailed analysis plan will be developed and agreed by members of the Collaboration prior to the collection and analysis of data from the individual trials.

### Eligibility criteria for studies to be included

Studies will be included if they are randomised trials. The level of allocation concealment within each trial will be assessed according to the criteria outlined in the Cochrane Handbook [42], and only those trials with adequate allocation concealment will be eligible. Participating trials are required to register on a publicly accessible trials registry before enrolment of the first patient [44].

### Participants

Participants in the eligible trials will be infants born before 28 weeks' gestation and enrolled within 24 hours of birth.

### Interventions

The intervention will be random assignment to either a lower (SpO_2 _85-89%) or higher (SpO_2 _91-95%) functional oxygen saturation target range from birth, or soon thereafter, for durations as specified in each trial protocol (see Table 2). Intervention assignment must be masked to parents, care-givers and outcome assessors by the use of pulse oximeters that have been adjusted to display either 3% above or below the infant's actual saturation value, within the 85-95% oxygen saturation range.

**Table 2:** Characteristics of randomised trials included in NeOProM Collaboration

***Trial acronym***	BOOST II-Australia	BOOST II-UK	BOOST-NZ	SUPPORT	COT
***Registration number***	ACTRN12605000055606	ISRCTN00842661	ACTRN12605000253606	NCT00233324	ISRCTN62491227
***Planned sample size***	1200	1200	320	1310	1200
***Countries of recruitment***	Australia	United Kingdom	New Zealand	United States	Canada, USA, Argentina, Germany, Israel, Finland
***Participants***	Infants < 28 wks gestation inborn or outborn < 24 hrs old	Infants < 28 wks gestation < 12 hrs old (24 hrs if outborn)	Infants < 28 wks gestation inborn or outborn < 24 hrs old	Infants 24-27 wks gestation < 2 hrs old	Infants 23 0/7-27 6/7 wks gestation < 24 hrs old
***Masked?***	Yes	Yes	Yes	Yes	Yes
***Intervention***	Lower oxygen saturation (85%-89%)	Lower oxygen saturation (85%-89%)	Lower oxygen saturation (85%-89%)	Lower oxygen saturation (85%-89%)	Lower oxygen saturation (85%-89%)
***Comparator***	Higher oxygen saturation (91%-95%)	Higher oxygen saturation (91%-95%)	Higher oxygen saturation (91%-95%)	Higher oxygen saturation (91%-95%)	Higher oxygen saturation (91%-95%)
***Intervention & comparator duration***	Oximeter applied asap after admission to NICU, continued for minimum 2 wks. Thereafter continued until 36 wks corrected age or SpO_2 _> 96% in room air for 95% of time over 3 days.	Oximeter applied from randomisation until postmenstrual age (PMA) of 36 wks or until baby is breathing air. All monitoring at any time prior to 36 wks to be done using study oximeter. BPD defined at 36 wks using a physiological test.	Oximeter applied asap after admission to NICU, continued for minimum 2 wks. Thereafter continued until 36 wks corrected age or SpO_2 _> 96% in room air for 95% of time over 3 days.	Oximeter applied within 2 hrs following admission to NICU until infant has been in room air for 72 hrs or until 36 wks corrected age, assessed by physiologic oxygen test.	Oximeter applied from day of birth until a minimum 36 wks PMA. If breathing room air without any form of respiratory assistance from 35 wks PMA onward, study oximetry discontinued at a 36 wks PMA. If receiving any form of respiratory assistance and/or oxygen therapy from 35 wks PMA onward study oximetry continues until 40 wks PMA. Study oximetry stopped at any time before 40 wks PMA if baby discharged home (with or without respiratory assistance and/or oxygen).
***Primary outcome(s)***	Death or survival with major disability at 2 yrs corrected for gestation. Major disability defined as having any of the following: * cognitive score <70 on BSID-3 * severe visual loss * cerebral palsy with inability to walk at 2 yrs * deafness requiring hearing aids	Death or survival with major disability at 2 yrs corrected for gestation. Major disability defined as having any of the following: * cognitive score <70 on BSID-3 * severe visual loss * cerebral palsy with inability to walk at 2 yrs * deafness requiring (or too severe to benefit from) hearing aids	Death or survival with major disability at 2 yrs corrected for gestation. Major disability is defined as having any of the following: * cognitive score <70 on BSID-3 * severe visual loss * cerebral palsy with inability to walk at 2 yrs * deafness requiring hearing aids	1. Death or survival with neurodevelopmental outcome at 18-22 mths corrected age. 2. Survival without severe ROP (threshold ROP and/or the need for surgical intervention)	Death or survival with major disability at 18-21 mths Major disability defined as having any of the following: * cognitive score <85 and/or language score <85 on BSID-3 * severe visual loss * cerebral palsy with inability to crawl or walk independently * deafness requiring hearing aids

### Outcomes

Analysis will be of all infants ever randomised and will be based on intention-to-treat. Binary outcomes will be analysed using log-binomial regression models adjusting for trial differences by including the trial variable in the model specification. Exponentiating the parameter estimate for treatment from a log-binomial regression model produces a relative risk for treatment. Continuous normally distributed endpoints will be analysed using a linear fixed effects model. Additionally the treatment by trial interaction will be assessed to investigate possible heterogeneity of treatment effects [48]. The overall estimated mean and standard deviation within each treatment group will be presented along with the mean difference in treatment effect and its 95% confidence interval with p value. If the data do not meet the assumptions for the model then transformations or alternative models will be investigated. If appropriate, multivariable regression modeling will be undertaken as exploratory analyses to assess treatment by covariate interactions. A detailed analysis plan will be developed as a separate document.

#### 1. Primary outcome

• composite outcome of death or major disability by 18-24 months corrected age (gestational age plus chronological age). Major disability is any of the following: Bayley III Developmental Assessment cognitive score <85 and/or language score <85 [49], severe visual loss (cannot fixate or is legally blind: <6/60 vision, 1.3 logMAR in both eyes) [50], cerebral palsy with Gross Motor Function Classification System (GMFCS) level 2 [51] or higher or Manual Ability Classification System (MACS) level 2 or higher [52] at 18-24 months postmenstrual age, or deafness requiring hearing aids.

#### 2. Additional outcomes

• ROP treatment by laser photocoagulation or cryotherapy (performed if Type I ROP or threshold ROP occurs) [14]

• measures of respiratory support, defined as (a) supplemental oxygen requirement at 36 weeks' postmenstrual age (see Table 2), (b) days of endotracheal intubation (c) days of continuous positive airway pressure (CPAP), (d) days of supplemental oxygen, (e) days on home oxygen

• patent ductus arteriosus diagnosed by ultrasound and requiring medical treatment

• patent ductus arteriosus requiring surgical treatment

• necrotising enterocolitis requiring surgery

• weight at birth, 36 weeks' postmenstrual age, discharge home and 18-24 months corrected age

• re-admissions to hospital up to 18-24 months corrected age

• cerebral palsy with GMFCS level 2 or higher or MACS level 2 or higher at 18-24 months corrected age

• blindness (<6/60 vision, 1.3 logMAR in both eyes)

• deafness requiring hearing aids

• quantitative Bayley III scores

• death

#### 3. Planned subgroup analyses

The effect of the intervention (higher versus lower oxygen saturation targeting) may be differential due to certain characteristics of either the infant or the way the intervention was delivered. These possible effects will be explored by the following subgroup analyses.

a. Patient baseline characteristics

• gestational age (<26 weeks/>26 weeks)

• inborn or outborn status

• antenatal steroids (any: yes/no)

• gender (male/female)

• small for gestational age (yes/no)

• multiples (singleton/multiple)

• mode of delivery (vaginal/caesarean)

b. Intervention characteristics

• time of intervention commencement (<6 hours/>6 hours)

• oximeter adjustment algorithm (original or revised)

#### 4. Planned sensitivity analyses

To assess whether results are robust to different methods of analysis and trial quality the following sensitivity analyses will be conducted:

• comparison of analyses using random effects and fixed effect models

• analysis of outcomes weighted by degree of oxygen saturation separation between treatment and control [53]

### Ethics and management issues

#### Search methods for identification of studies

Efforts to identify any ongoing trials that may be eligible for participation in this PMA included searches for published protocols on online databases such as Medline and Embase as well as internet searches for non-peer reviewed articles and other publications using Google. Further efforts included informing networks of the proposed PMA and approaching presenters at relevant conferences and network meetings. Ongoing trials will only be permitted to join the PMA prior to the results of any of the participating trials being made publicly available.

#### Data monitoring procedures

Because different oxygen targets may have competing risks, it is essential that sufficiently large numbers of recruits across all trials are allowed to accumulate to be able to demonstrate net clinical benefit or harm. Each participating trial will have its own Data and Safety Monitoring Committee (DSMC). The Principal Investigators of each trial will seek to ensure that the Chairperson of their own DSMC knows of the existence of the other participating trials and their DSMC Chairpersons so that communication can occur between them if required. The NeOProM Management Committee will give consideration to any requests from DSMC Chairpersons for sharing of de-identified data (either aggregate or individual patient data) should the need arise. There are 'in principle' plans to update the PMA data at regular intervals as longer term follow-up data become available but (as of December 2009) there are no formalised plans yet agreed.

#### Project management

Membership of the NeOProM Collaboration includes representative(s) from each of the trials contributing data to the project with an accompanying project coordination and data management structure. An international Steering Group has been established with all collaborating trials being represented as well as experts in the fields of PMA, IPD meta-analysis, data monitoring and trial compliance and design being included. Project coordination and data management/analysis are coordinated from the NHMRC Clinical Trials Centre, University of Sydney, Australia.

#### Funding

Funding for the NeOProM Collaboration has, and will continue to be, sought from relevant funding agencies. Each individual trial is receiving funding from their own respective national government research funding bodies, including the National Institutes of Health (United States), National Health and Medical Research Council (Australia), Health Research Council (New Zealand), Medical Research Council (United Kingdom) and Canadian Institutes of Health Research (Canada).

#### Publication policy

Each of the participating trials will be able to publish their main individual trial results prior to publication of the final PMA results. Each of the participating trials will seek to include reference to the NeOProM Collaboration in the published abstract and, if possible, in the text of their main individual trial publication. The main manuscript will be prepared by the NeOProM Steering Group, before circulation to the full Collaborative Group for comment and revision. Publications using these data will be authored on behalf of the NeOProM Collaboration, either with specific named authors, or on behalf of the Collaboration as a whole, as agreed by the Steering Group. Names of other participating Collaborators will be acknowledged in an appropriate section of the manuscript. Subsequent analyses and publications will only be undertaken via collaboration with, and with the approval of, the NeOProM Collaboration Steering Group.

#### Ethical considerations

Parents of participants in the individual trials have previously consented to participation by their children in their respective trial. The data for this project are to be used for the purpose for which they were originally collected and are available through an agreement between all trialists of the NeOProM Collaboration. These trialists remain the custodians of their original individual trial data at all times and have the right to withdraw some or all of their data from the analyses. Data are provided on the stipulation that all trials have received ethical clearances from their relevant bodies.

## Discussion

Results should be available by 2014.

## Abbreviations

NeOProM: Neonatal Oxygenation Prospective Meta-analysis; PMA: prospective meta-analysis; ROP: retinopathy of prematurity; SpO_2_: arterial oxygen saturation; PaO_2_: arterial oxygen tension; NICU: Neonatal Intensive Care Unit; RRR: relative risk ratio; IPD: individual patient data; GMFCS: Gross Motor Function Classification System; MACS: Manual Ability Classification System; DSMC: Data and Safety Monitoring Committee.

## Competing interests

The authors declare that they have no competing interests.

## Authors' contributions

This protocol document was developed by the authors in consultation with members of the NeOProM Collaboration as listed in the Acknowledgements section below. All members of this group were sent draft versions and invited to comment and contribute changes. All authors read and approved the final manuscript.

## Pre-publication history

The pre-publication history for this paper can be accessed here:

http://www.biomedcentral.com/1471-2431/11/6/prepub
